# Key Aspects in the Nutritional Management of Polycystic Liver Disease Patients

**DOI:** 10.3390/nu17142380

**Published:** 2025-07-21

**Authors:** Saniya Khan, Simone Di Cola, Silvia Lai, Flaminia Ferri, Vincenzo Cardinale, Manuela Merli

**Affiliations:** 1 Department of Translational and Precision Medicine, Sapienza University of Rome, 00185 Rome, Italy; saniya.khan@uniroma1.it (S.K.); simone.dicola@uniroma1.it (S.D.C.); flaminia.ferri@uniroma1.it (F.F.); 2 Nephrology Unit, Department of Translational and Precision Medicine, Sapienza University of Rome, 00185 Rome, Italy; silvia.lai@uniroma1.it; 3 Beth Israel Deaconess Medical Center, Division of Gastroenterology, Hepatology and Nutrition, Harvard Medical School, Boston, MA 02215, USA

**Keywords:** polycystic liver disease, nutrition, malnutrition, sarcopenia, quality of life

## Abstract

Polycystic liver disease (PLD) is a rare genetic disorder characterized by the development of >10 fluid-filled cysts in the liver. While PLD can occur in isolation, it is most commonly associated with autosomal dominant polycystic kidney disease, adding complexity to its management. PLD is often asymptomatic but can lead to hepatomegaly, causing symptoms such as abdominal distension, pain and discomfort, early satiety, gastroesophageal reflux, and malnutrition, ultimately affecting patients’ quality of life. Current treatment strategies, including pharmacological and interventional approaches, focus on reducing liver volume and alleviating symptoms. However, management remains largely symptomatic, as no definitive therapies exist to halt cyst progression. Liver transplantation is the only curative option for patients with severe, progressive disease and refractory complications. The EASL guidelines recognize that PLD-related symptoms, primarily due to hepatomegaly, can contribute to involuntary weight loss and recommend assessing symptomatic patients for malnutrition and sarcopenia. Although evidence suggests that patients with PLD may be at risk of malnutrition, original data on the quality and extent of nutritional alterations remain scarce. The potential influence of nutrition on disease progression, symptom burden, and overall well-being is also largely unexplored. Given these knowledge gaps, addressing nutritional challenges, such as early satiety, is essential for optimizing symptom management and maintaining overall nutritional status. This review outlines a possible pathophysiology of malnutrition, specific dietary considerations and recommendations, and weight management in patients with PLD. Additionally, dietary complexities in patients with concurrent renal involvement are discussed, offering a practical framework for clinicians and dietitians in managing this challenging condition.

## 1. Introduction

Polycystic liver disease (PLD) is a rare genetic disorder characterized by progressive development of multiple fluid-filled cysts in the liver for inherent ciliopathy and cholangiopathy, leading in some cases to hepatomegaly and, in severe forms, significant clinical complications [1,2]. Although PLD can occur in isolation as autosomal dominant PLD (ADPLD), it is more commonly associated with autosomal dominant polycystic kidney disease (ADPKD), which complicates its management and prognosis [3].

ADPLD affects 1/100,000 individuals worldwide, while ADPKD has a prevalence of 1/1000 people; a rare form of autosomal recessive polycystic kidney disease (ARPKD) affects 1/10,000 to 1/40,000 individuals [4] and is associated with congenital hepatic fibrosis rather than liver cysts (PKHD1, DZIP1L, CYS1, PKD1) [5]. The etiology is multifactorial and involves genetic, epigenetic, hormonal, cell signaling abnormalities, overexpression of profibrogenetic proteins, and alterations in bile acid metabolism [6,7,8,9].

Although the literature frequently reports the significant risk of malnutrition in PLD, original data about the quality and quantity of nutritional alterations remain scanty. The potential influence of nutrition on disease progression, symptom burden, and overall well-being also remains largely underexplored. Given these limitations, supportive care, including targeted nutritional management, could be beneficial in addressing symptoms like early satiety and maintaining nutritional status.

Guidelines from the European Association for the Study of the Liver (EASL) and Kidney Disease Improving Global Outcomes (KIDGO) emphasize the importance of assessing malnutrition and sarcopenia in symptomatic PLD. CT imaging, when available, is recommended to simultaneously assess sarcopenia and the displacement of adjacent organs (e.g., stomach and intestines) due to massive hepatomegaly. The EASL further recommends that patients with PLD and sarcopenia undergo nutritional assessment and management under the supervision of dietitians [5,10].

PLD-related symptoms, particularly involuntary weight loss, are largely attributable to hepatomegaly. Guidelines advocate for a multidisciplinary approach, incorporating nutritional and physiotherapeutic counselling alongside symptom management. This integrated strategy may yield synergistic benefits, improving patient-reported outcomes such as disease-specific symptom scores [11].

This review seeks to explore the pathophysiology of malnutrition in PLD, and to outline the dietary considerations and recommendations for improving and/or maintaining nutritional status and managing symptoms in patients with PLD. The focus is exclusively on the nutritional aspect, and more extensive reviews about pathophysiology, clinical aspects, and the treatment of PLD are already present in the literature. Additionally, it also addresses the dietary complexities in those with concomitant renal involvement, providing a practical framework for clinicians and dietitians in managing this challenging condition. To our knowledge, this is the first review dedicated specifically to nutrition in PLD.

## 2. Polycystic Liver Disease Symptoms and Current Treatment

PLD is often asymptomatic early on; however, as cysts enlarge and multiply, many patients experience significant abdominal distension, early satiety, nausea, gastroesophageal reflux, and, in severe cases, dyspnea and malnutrition due to the massive hepatic enlargement, all of which have a profound impact on patients’ quality of life [12,13]. A PLD questionnaire (PLDQ) can be used to assess the frequency and discomfort of PLD-associated symptoms [14]. In patients with ADPKD, the most common pressure-related symptoms were back and flank pain, abdominal fullness, dyspnea, mass sensation, and early satiety. These symptoms were directly correlated with the severity of the ADPKD, as measured by the height-adjusted total liver volume (HTLV). Kim H. and colleagues found that an HTLV of over 1600 mL/m^2^ was associated with a fivefold increase in the risk of pressure-related symptoms [15].

Current treatment focuses on liver volume reduction and symptom relief. Several clinical trials have provided evidence that somatostatin analogues may help reduce cyst growth, particularly in cases of numerous small to medium cysts throughout the liver [10]. According to international guidelines, liver resection is indicated in selected cases of PLD with clustered cysts localized to a few liver segments and preservation of relatively unaffected parenchyma. For large symptomatic cysts, interventional procedures such as cyst aspiration, sclerotherapy and fenestration are used [4]. All these therapeutic approaches, however, are not recommended for diffuse or massive PLD. Despite this, management remains largely symptomatic, and there are no definitive therapies to halt cyst growth. A liver transplant is the only definitive treatment for patients with severe, progressive PLD who develop severe complications unresponsive to other therapeutic options or, rarely, liver failure [10]. This highlights the need for supportive strategies, particularly nutritional intervention, to help preserve functional status and quality of life.

## 3. Materials and Methods

### 3.1. Methodology for the Literature Search

We conducted a comprehensive literature search in PubMed, Web of Science, and Cochrane Library from inception until February 2025 to identify studies related to nutrition and PLD. The search strategy was as follows: polycystic liver disease [MeSH terms] AND (nutrition OR malnutrition OR muscle mass OR sarcopenia OR muscle OR body weight OR protein OR energy OR BMI). Both original research articles and reviews were included.

Our search did not identify any original study specifically addressing nutritional assessment and management in patients with PLD. Therefore, the information presented is derived from review studies discussing the overall management of PLD, where malnutrition is often mentioned.

### 3.2. Methodology for the Cohort Data

#### 3.2.1. Study Population

In this cross-sectional observational study, stable adult patients with a diagnosis of PLD attending the Outpatient Clinic at Policlinico Umberto I, Sapienza University of Rome, from July 2023 until May 2025 underwent a nutritional screening. The exclusion criteria were: (1) age < 18 years or >75 years; (2) inability to undergo the screening tests; (3) glomerular filtration rate (eGFR) < 30 mL/min; (4) advanced cardiac or pulmonary disease (NYHA III-IV, GOLD > 3); and (5) patients with neuromuscular and psychiatric disorders.

#### 3.2.2. Ethical Approval

The study was approved by the Ethical Committee of the Sapienza University of Rome (Prot. 0057/2023 approved on 26 January 2023). Informed consent was obtained from all participants.

#### 3.2.3. Data Collection

Demographic and clinical information, including sex, age, previous clinical history, biochemical and radiological parameters, and relevant comorbidities were recorded from patients’ hospital records.

#### 3.2.4. Nutritional Assessment

Body mass index (BMI) was calculated using a standard formula. BMI was categorized as follows: underweight or malnourished (<18.5 kg/m^2^), normal weight (18.5–24.9 kg/m^2^), and overweight/obese (>25 kg/m^2^).

Dietary habits were assessed using a 24-h dietary recall (24 HR), which was employed to estimate the daily energy and protein intake.

#### 3.2.5. Physical and Muscle Mass Assessment

Mid-upper arm circumference (MUAC) was measured with a non-stretchable tape with an accuracy of 0.1 cm. Triceps skinfold (TSF) thickness was measured using Harpenden calipers with an accuracy of 1 mm at the mid-point between the acromion and the olecranon processes. All the measurements were taken on the non-dominant arm.

Handgrip strength (HGS) was measured in kilograms using a handheld dynamometer in the dominant hand, with an average of three trials used for analysis [16]. Low muscle function was defined as HGS < 27 kg in males and <16 kg in females [17]. Physical performance was further assessed using a balance test, where participants balanced for up to 10 s in three positions (side-by-side, semi-tandem, and tandem), and the chair stand test (CST), which timed the completion of five chair stands with arms folded across the chest [18].

#### 3.2.6. Statistical Analysis

Statistical analysis was performed using SPSS software (version 28.0.1.1). Data were expressed as mean ± standard deviation (SD) in the case of continuous variables and as number (percentages) for categorical variables.

## 4. The Role of Nutrition in Polycystic Liver Disease

### 4.1. The Effect of Diet on Liver Health

Nutrition plays a critical role in preserving liver function and slowing the progression of liver diseases. The liver’s central role in metabolism makes it highly sensitive to the quality and quantity of dietary intake. Excessive consumption of saturated fats, refined sugars, and alcohol contributes to fat accumulation, oxidative stress, and systemic inflammation, which can lead to conditions like metabolic-associated steatotic liver disease (MASLD), cirrhosis, and hepatocellular carcinoma [19]. Conversely, diets rich in antioxidants, such as vitamins C and E, polyphenols, and omega-3 fatty acids, may reduce inflammation and oxidative stress, protecting hepatocytes from injury. Low-glycemic index carbohydrates help regulate blood sugar levels, reducing insulin resistance [20], which is a common contributor to liver dysfunction [21]. Weight management through caloric control and regular physical activity (aerobic and resistance) is crucial, as obesity contributes to liver damage [22].

### 4.2. Nutritional Status in Patients with Polycystic Liver Disease

Patients with PLD are at a significant risk of malnutrition due to the mechanical effects of hepatic cyst enlargement on the gastrointestinal system [23]. The progressive increase in liver volume often leads to gastric and duodenal compression, resulting in early satiety, postprandial fullness/discomfort, digestive intolerance, and gastroesophageal reflux [3,23,24,25]. These symptoms contribute to a marked reduction in dietary intake, further exacerbating nutritional deficiencies. Moreover, abdominal distention, pain, and increased abdominal pressure can suppress appetite and further reduce food intake [24].

**Figure 1:**
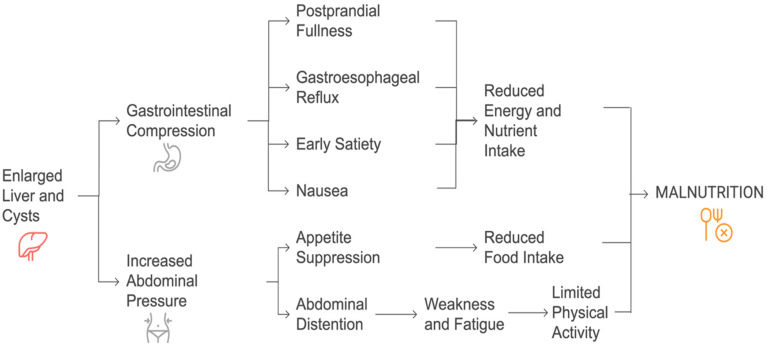
Proposed pathophysiology of malnutrition in polycystic liver disease (PLD).

The development of cysts in the left liver or segment I can lead to compression of the gastric or duodenal regions, even in the presence of moderate hepatomegaly. Initially, this may cause patients to limit their number of meals, and as the condition worsens, they might voluntarily reduce their food intake to ease the discomfort [24]. Prolonged food restriction, coupled with decreased physical activity due to abdominal discomfort and recurrent hospitalization, can result in muscle wasting and hypoproteinemia, ultimately affecting their quality of life [26,27]. Abdominal distention, resulting in increased abdominal weight, further suppresses movement, contributing to muscle depletion. Unlike malnutrition in liver cirrhosis, which is primarily linked to hepatocellular insufficiency and metabolic disturbances, PLD-related malnutrition stems from mechanical compression of the gastrointestinal tract and reduced nutrient intake [28]. Studies indicate that patients with severe hepatomegaly often experience protein–energy malnutrition, which worsens as the disease progresses [29]. A proposed pathophysiology has been outlined in Figure 1.

The progressive enlargement of liver cysts can contribute to hepatomegaly and abdominal distension, partially masking weight loss and delaying the diagnosis of malnutrition. To accurately assess nutritional status in these patients, a comprehensive approach is essential, including clinical examination, laboratory tests, and, when available, CT imaging of subcutaneous fat and muscle mass [24].

Specific considerations apply to the pathogenesis of malnutrition and sarcopenia in patients with PLD-related complications, including jaundice, hepatic venous outflow obstruction, portal hypertension, recurrent cyst infections, and recurrent cyst hemorrhage. These complications may further contribute to the development of malnutrition and sarcopenia [10,30].

### 4.3. Nutritional Goals for Patients with Polycystic Liver Disease

Although there is limited research on specific foods that directly impact liver cyst growth in PLD, avoiding pro-inflammatory and hepatotoxic foods is a reasonable approach. For patients with PLD, nutritional management may focus on supporting overall liver health while addressing the specific challenges posed by the disease. Maintaining an appropriate energy balance is essential to avoid unintentional weight loss or gain, both of which can strain the liver. High-protein diets are particularly beneficial as they prevent/reduce muscle loss and improve nitrogen balance [31]. Emphasizing anti-inflammatory foods, such as leafy greens, berries, nuts, seeds, and fatty fish, can help reduce hepatic inflammation and cyst growth [32]. Minimizing ultra-processed foods that are often high in sodium, unhealthy fats, and added sugars, provides a foundation for effective dietary management [33]. Alcohol can accelerate liver damage, refs. [34,35] making moderation or complete abstinence advisable for patients with PLD. Additionally, excessive sugar intake, particularly from fructose-sweetened beverages and refined carbohydrates, has been linked to hepatic steatosis and insulin resistance, both of which may worsen liver-related complications [36].

### 4.4. Specific Dietary Challenges Faced by Polycystic Liver Disease Patients

Patients with PLD often deal with significant dietary challenges stemming from the physical effects of liver cysts. Enlarged cysts can compress the stomach and intestines, leading to early satiety, reduced appetite, and difficulty tolerating large meals [28]. This requires tailored dietary adjustments, such as consuming small, frequent meals that are rich in nutrients and energy. Protein–energy malnutrition is another concern, necessitating the inclusion of high-quality protein sources like lean meats, eggs, and dairy products. Additionally, fiber-rich foods should be balanced to support digestion without worsening abdominal discomfort. Multivitamin supplements, including fat-soluble vitamins (A, D, E, K), may be required in cases of malabsorption or advanced disease [30]. Table 1 summarizes the possible mechanisms, consequences, and strategies for managing malnutrition in PLD.

## 5. Dietary Recommendations for Polycystic Liver Disease Patients

### 5.1. Dietary Sodium

Patients suffering from PLD may develop hepatic venous outflow obstruction, portal vein obstruction, and/or inferior cava vein syndrome because of the cystic mass effect. This can cause portal hypertension, although rare, leading to ascites, variceal hemorrhage, or splenomegaly [37]. In patients with chronic liver diseases such as cirrhosis, a low-sodium diet is a cornerstone to prevent fluid retention and control blood pressure, with targets typically set at less than 2000 mg of sodium per day (5 g or 1 teaspoon salt) [30]. Studies and guidelines underscore the benefit of reduced sodium intake in minimizing ascites and edema [30,38,39], which are complications that can also affect patients with PLD due to mass effects from enlarged cysts [37]. However, it is important to consider that aggressive salt restriction (<5 g/day) can make food unpalatable, leading to altered dietary habits and potentially contributing to malnutrition [40,41,42]. Severe salt restriction could also lead to hyponatremia, which might adversely affect kidney function [41].

A possible indication in PLD patients with ascites is to significantly reduce the consumption of ultra-processed foods and use of herbs and spices in place of excess salt to enhance flavor, wherever necessary.

### 5.2. Macronutrient Considerations (Proteins, Fats, Carbohydrates)

Macronutrient balance is essential to prevent malnutrition. A daily intake of 1.2–1.5 g/kg body weight of high-quality protein is advised to counteract muscle wasting in patients with chronic liver disease [39]. Lean proteins are emphasized to optimize nitrogen balance without overburdening hepatic metabolism. Regarding fats, the focus is on unsaturated fats from sources like olive oil, avocados, and nuts, which are beneficial for cardiovascular health and may exert anti-inflammatory effects, whereas saturated and trans fats are limited to reduce hepatic stress [43,44,45]. Carbohydrate recommendations stress the importance of complex, fiber-rich sources (whole grains, fruits, and vegetables) to ensure steady energy release and maintain glycemic control, thereby minimizing the risk of insulin resistance, which is a known contributor to liver fat accumulation [20].

Specific considerations are needed for patients with PLD and associated polycystic kidney disease (PKD). The presence of both these conditions can complicate clinical management, as PKD can lead to progressive renal insufficiency or kidney failure. As kidney function declines, managing fluid and electrolyte balance becomes more challenging, especially with the kidneys’ reduced ability to handle excess fluid and sodium [46].

In patients with impaired kidney function, maintaining proper fluid and salt balance is crucial. Fluid retention and electrolyte imbalances can occur, which may increase the risk of hypertension and worsen kidney function [46]. Furthermore, in patients with both PLD and PKD, dietary recommendations must be carefully tailored. A hyperproteic diet may not be ideal for those with reduced kidney function. A high-protein diet can stimulate glucagon secretion, gluconeogenesis, and urea excretion, which, along with vasopressin release, leads to increased water reabsorption and glomerular hyperfiltration. This process can contribute to accelerated kidney damage and cystogenesis [47,48]. Intake of animal-sourced protein has been shown to influence cyst growth in ADPKD [49]. The Consortium for Radiologic Imaging of PKD (CRISP) study found that ADPKD patients with the highest urea excretion, an indicator of protein intake, had larger kidneys that progressed more rapidly to renal failure [49,50]. The disease phenotype varies significantly, leading to different rates of cyst growth even among family members [51]. Dietary factors, particularly excessive intake of salt and animal protein, contribute to this variability and may accelerate kidney enlargement [50,52]. Therefore, protein intake should be carefully adjusted based on the stage of renal function, ensuring any restriction is accompanied by adequate energy intake to prevent malnutrition.

## 6. Overweight and Obesity in Polycystic Liver Disease

Malnutrition is the primary concern in PLD, however a subset of patients may present as overweight or obese. In these patients, excess adiposity may contribute to increased intra-abdominal pressure, worsening symptoms such as early satiety, bloating, and discomfort due to an enlarged liver. Weight management, when indicated, should focus on gradual, sustainable lifestyle changes and dietary strategies Moderate calorie restriction should be combined with adequate protein intake (1.2–1.5 g/kg body weight/day) to preserve lean body mass while supporting metabolic health [39]. Regular monitoring is essential to avoid unintended excessive weight loss and ensure nutritional adequacy.

In addition to dietary modifications, regular physical activity plays a key role in weight management, preserving muscle mass, and improving metabolic function [53,54]. Abdominal bracing core exercises have been shown to be effective in reducing pain and increasing soft lean mass in ADPKD patients who have significant PLD [55]. For patients with PLD, low-impact exercises such as walking, swimming, and yoga may be preferable to minimize the discomfort caused by an enlarged liver.

## 7. Monitoring and Adjusting Nutrition Plans over Time

Patients with worsening liver function or symptoms such as early satiety, bloating, or fatigue may require tailored dietary adjustments. In particular, patients with large hepatic cysts that compress the stomach and displace other abdominal organs are at a significant risk for malnutrition and nutritional deficiencies [23]. A serious complication of PLD is massive hepatomegaly, which can lead to severe malnutrition—characterized by weight loss exceeding 10%—and may even be life-threatening [23]. Importantly, muscle wasting and marked nutritional deterioration are typically confined to patients with a severe disease phenotype. If signs of malnutrition or muscle loss (sarcopenia) emerge, protein intake may need to be increased (1.2–1.5 g/kg body weight/day), alongside ensuring adequate caloric intake to preserve muscle mass [39]. Key parameters such as liver enzyme levels (ALT, AST, ALP, GGT), bilirubin, albumin, markers of metabolic health (e.g., glucose, insulin resistance, lipid profile), and kidney parameters in the case of concurrent ADPKD should be monitored periodically to detect any changes that may necessitate dietary modifications.

Patients with PLD are rarely listed for LT due to liver failure [56]. LT is particularly indicated in patients with severe, disabling symptoms that significantly impair quality of life and result in severe malnutrition. While there are no standardized LT listing criteria for these patients, in Europe, severe malnutrition (defined as a mid-arm circumference of the non-dominant arm of less than 23.8 cm for males and less than 23.1 cm for females) qualifies as a MELD exception in most centers [57].

Since most patients with PLD are listed for LT through a MELD exception, it is advisable to assign additional points to the MELD score based on the severity of the complications to ensure proper prioritization. Factors such as total kidney and liver volume may further influence nutritional status, highlighting the importance of comprehensive nutritional assessment and intervention prior to transplantation [58]. Maintaining adequate preoperative nutritional status is crucial, as malnutrition and sarcopenia are associated with increased postoperative complications and mortality [59]. Therefore, optimizing nutrition before LT can reduce the risk of transplant list dropout, improve surgical outcomes, enhance recovery, and reduce the risk of post-LT complications.

A retrospective study in patients with PLD and polycystic liver and kidney disease reported a marked improvement in quality of life following transplantation, with resolution of abdominal fullness, pain, and malnutrition in cases of combined kidney and liver transplants [3]. However, quality of life was not systematically evaluated using validated tools, making it difficult to quantify the reported improvements or to identify which specific aspects of quality of life were affected. Similarly, a case report in a patient with PLD and PKD demonstrated improvements in dietary intake, nutritional status, physical activity, and consequently, albumin levels following LT [28]. Notably, neither study specified the assessment tools used, limiting the interpretability and reproducibility of these findings.

## 8. Observational Data from Our Outpatient Cohort

At our referral center, 17 outpatients with stable PLD underwent an evaluation of their nutritional status, and informed consent for data collection was obtained from all. The mean age was 57 ± 10.2 years, with the majority being female and 70% having concurrent PKD. The liver function test results of all patients were within normal range (Table 2) and none were under consideration for LT at the time of evaluation.

A reduced dietary intake was observed, with an average energy intake of 24.2 kcal/kg/day and protein intake of 0.89 g/kg/day, plausibly due to symptoms such as nausea (2, 11.7%), anorexia (4, 23.5%), and abdominal pain (7, 41%). Notably, 12 (70.5%) patients had energy intake below 25 kcal/kg/day. Given that the recommended protein intake for patients with chronic conditions is 1.2–2.0 g/kg/day, the observed intake appears insufficient, despite the absence of PLD-specific nutritional guidelines. A total of 13 (76.5%) patients had protein intakes of less than 1 g/kg/day. Two patients (11.7%) reported mild daily intake of alcohol (1 unit of alcohol/day).

Despite the reported reduction in intake, muscle function remained largely preserved. The mean HGS was 23.7 ± 7.3 kg, with only one female patient demonstrating low muscle strength. The mean time for CST was 11.6 ± 5.1 s, and all patients completed the balance test without any difficulty. MUAC and TSF were within the normal range. The mean BMI was 22.6 ± 4.4 kg/m^2^, with the majority of the patients falling within the normal BMI range (18.9–24.9 kg/m^2^) (Table 3).

Overall, clinical signs of malnutrition or sarcopenia were minimal in this cohort. It is possible that nutritional concerns primarily arise in those with a more severe disease phenotype, where extensive hepatomegaly can cause mechanical compression and symptoms such as early satiety. Our cohort data have some limitations. It included only stable outpatients who were not under consideration for LT; therefore, the prevalence of malnutrition in this cohort may not be generalizable. Another limitation is the use of 24 HR to assess dietary intake, which relies on self-reported data, is subjective, and is susceptible to recall bias. Factors such as patients’ memory, comprehension, and willingness to report accurately may affect the reliability and validity of the data. Nonetheless, routine nutritional screening and timely dietary interventions is relevant to prevent future nutritional decline. Clinical management should be guided by symptom burden, with attention to nutritional support primarily in those exhibiting advanced disease characteristics. While the size and location of cysts may contribute to the degree of gastric compression that limits food intake, most patients do not develop deficiencies until the later stages. However, observational studies with larger sample sizes are needed to better understand the nutritional profile of this population. Subsequently, nutritional intervention trials should be designed and implemented.

## 9. Conclusions

PLD presents unique nutritional challenges, especially in patients exhibiting a severe disease phenotype. The progressive enlargement of hepatic cyst causes mechanical compression of the gastrointestinal tract, which often leads to reduced dietary intake and can eventually pose a threat for the development of malnutrition and sarcopenia. Unlike other chronic liver diseases where malnutrition largely stems from metabolic dysfunction, PLD-related malnutrition results from symptom-driven reduced food intake and physical inactivity. Optimal nutritional management for PLD should focus on maintaining adequate energy and high-quality protein intake to preserve muscle mass. Special attention is warranted in cases with concurrent polycystic kidney disease, where protein and sodium intake must be carefully balanced to prevent further renal damage. Regular monitoring and individualized dietary adjustments are essential to address evolving nutritional needs and prevent deterioration. Although liver transplantation remains the definitive treatment for advanced PLD with severe symptoms and malnutrition, early nutritional intervention might play a supportive role in managing the condition by improving quality of life and potentially delaying nutritional decline. Further research is needed to establish evidence-based dietary guidelines specific to PLD and to better understand the impact of nutrition in this patient group.

## Figures and Tables

**Table 1:** Mechanisms, consequences, and strategies for managing malnutrition in polycystic liver disease.

Cause	Consequence	Possible Management Strategy
Voluminous cysts	Reduced gastric capacity leading to early satiety	Reduce compression by cyst(s) with intervention (cyst aspiration, sclerotherapy, or surgical intervention)
Gastrointestinal compression	Postprandial fullness, gastroesophageal reflux, nausea	Provide small, frequent, energy-dense meals; prokinetic agents if indicated
Abdominal compression	Fluid retention, ascites	Recommend low-sodium diet and minimize ultra-processed foods; avoid severe salt restriction
Increased abdominal pressure	Suppressed appetite	Ensuring adequate caloric and protein intake via small, frequent meals
Abdominal distension	Reduced physical activity leading to muscle wasting, weakness	Suggest tailored physical activity program, including resistance training and physiotherapy
Pain or discomfort from cysts	Decreased food intake, sleep disturbances	Adequate pain management, positioning strategies to relieve pressure
Nutritional deficiencies (e.g., fat-soluble vitamins, minerals)	Compromised immune function	Prescribe micronutrient supplementation based on individual patient assessment
Hepatic dysfunction (if present)	Altered metabolism, protein–energy malnutrition	Advise high-protein diet (unless contraindicated), supplementation if needed
Concurrent ADPKD with renal involvement	Risk of electrolyte imbalances (e.g., potassium, phosphorus), fluid overload	Monitor renal function regularly; adjust protein, potassium, and phosphorus intake, as per renal status; adequate hydration as tolerated

**Table 2:** Demographic, clinical, and radiological characteristics of 17 patients with a diagnosis of polycystic liver disease.

Variables	All (*n* = 17)
Age (years)	57 ± 10.2
Gender (female)	16 (94)
Concurrent PKD	12 (70.5)
Co-morbidities	7 (41.1)
Total bilirubin (mg/dL)	0.6 ± 0.3
AST (IU/L)	23 ± 6.1
ALT (IU/L)	21 ± 5.8
ALP (IU/L)	70 ± 30.2
Total protein (g/dL)	7.1 ± 0.5
Serum albumin (g/dL)	4.2 ± 0.3
Creatinine (mg/dL)	1.3 ± 1.1
Right liver lobe size (cm)	16.9 ± 3.5
Maximum cyst size (cm)	6.4 ± 3

Data represented as mean ± SD or number (percentage). Abbreviations: PKD—polycystic kidney disease; AST—aspartate transaminase; ALT—alanine aminotransferase; and ALP—alkaline phosphatase.

**Table 3:** Nutritional status and dietary intake of 17 patients with a diagnosis of polycystic liver disease.

Variables.	All (*n* = 17)
Energy intake (kcal/kg/day)	24.2 ± 5
Protein intake (g/kg/day)	0.89 ± 0.16
HGS (kg)	23.7 ± 7.3
CST (s)	11.6 ± 5.1
MUAC (cm)	28 ± 4
TSF (mm)	14.5 ± 5.5
BMI (kg/m^2^) Malnourished (<18.5 kg/m^2^) Normal (18.5–24.9 kg/m^2^) Overweight/obese (>25 kg/m^2^)	22.6 ± 4.4 3 (17.6) 9 (53) 5 (24.9)

Data represented as mean ± SD or number (percentage). Abbreviations: HGS—handgrip strength; CST—chair stand test; MUAC—mid-upper arm circumference; TSF—triceps skinfold; and BMI—body mass index.

## References

[1] Roediger R., Dieterich D., Chanumolu P., Deshpande P. (2022). Polycystic Kidney/Liver Disease. Clin. Liver Dis..

[2] Torres V.E., Harris P.C., Pirson Y. (2007). Autosomal Dominant Polycystic Kidney Disease. Lancet.

[3] Zhang Z.-Y., Wang Z.-M., Huang Y. (2020). Polycystic Liver Disease: Classification, Diagnosis, Treatment Process, and Clinical Management. World J. Hepatol..

[4] Norcia L.F., Watanabe E.M., Filho P.T.H., Hasimoto C.N., Pelafsky L., de Oliveira W.K., Sassaki L.Y. (2022). Polycystic Liver Disease: Pathophysiology, Diagnosis and Treatment. Hepatic Med. Evid. Res..

[5] Torres V.E., Ahn C., Barten T.R.M., Brosnahan G., Cadnapaphornchai M.A., Chapman A.B., Gall E.C.-L., Drenth J.P.H., Gansevoort R.T., Harris P.C. (2025). KDIGO 2025 Clinical Practice Guideline for the Evaluation, Management, and Treatment of Autosomal Dominant Polycystic Kidney Disease (ADPKD): Executive Summary. Kidney Int..

[6] Wong M.Y., McCaughan G.W., Strasser S.I. (2017). An Update on the Pathophysiology and Management of Polycystic Liver Disease. Expert Rev. Gastroenterol. Hepatol..

[7] Aapkes S.E., Bernts L.H.P., Barten T.R.M., van den Berg M., Gansevoort R.T., Drenth J.P.H. (2021). Estrogens in Polycystic Liver Disease: A Target for Future Therapies?. Liver Int. Off. J. Int. Assoc. Study Liver.

[8] Cordido A., Vizoso-Gonzalez M., Nuñez-Gonzalez L., Molares-Vila A., Chantada-Vazquez M.D.P., Bravo S.B., Garcia-Gonzalez M.A. (2022). Quantitative Proteomic Study Unmasks Fibrinogen Pathway in Polycystic Liver Disease. Biomedicines.

[9] Alvaro D., Alpini G., Onori P., Franchitto A., Glaser S.S., Sage G.L., Folli F., Attili A.F., Gaudio E. (2002). Alfa and Beta Estrogen Receptors and the Biliary Tree. Mol. Cell. Endocrinol..

[10] Drenth J., Barten T., Hartog H., Nevens F., Taubert R., Balcells R.T., Vilgrain V., Böttler T. (2022). EASL Clinical Practice Guidelines on the Management of Cystic Liver Diseases. J. Hepatol..

[11] Billiet A., Temmerman F., Coudyzer W., den Ende N.V., Colle I., Francque S., Maeght S.D., Janssens F., Orlent H., Sprengers D. (2023). Questionnaire PLD-Complaint-Specific Assessment Identifies Need for Therapy in Polycystic Liver Disease: A Multi-Centric Prospective Study. United Eur. Gastroenterol. J..

[12] Qian Q., Li A., King B.F., Kamath P.S., Lager D.J., Huston J., Shub C., Davila S., Somlo S., Torres V.E. (2003). Clinical Profile of Autosomal Dominant Polycystic Liver Disease. Hepatology.

[13] Neijenhuis M.K., Kievit W., Verheesen S.M.H., D’Agnolo H.M., Gevers T.J.G., Drenth J.P.H. (2018). Impact of Liver Volume on Polycystic Liver Disease-Related Symptoms and Quality of Life. United Eur. Gastroenterol. J..

[14] Neijenhuis M.K., Gevers T.J.G., Hogan M.C., Kamath P.S., Wijnands T.F.M., van den Ouweland R.C.P.M., Edwards M.E., Sloan J.A., Kievit W., Drenth J.P.H. (2016). Development and Validation of a Disease-Specific Questionnaire to Assess Patient-Reported Symptoms in Polycystic Liver Disease. Hepatol. Baltim. Md..

[15] Kim H., Park H.C., Ryu H., Kim K., Kim H.S., Oh K.-H., Yu S.J., Chung J.W., Cho J.Y., Kim S.H. (2015). Clinical Correlates of Mass Effect in Autosomal Dominant Polycystic Kidney Disease. PLoS ONE.

[16] Fried L.P., Tangen C.M., Walston J., Newman A.B., Hirsch C., Gottdiener J., Seeman T., Tracy R., Kop W.J., Burke G. (2001). Frailty in Older Adults: Evidence for a Phenotype. J. Gerontol. Ser. A.

[17] Dodds R.M., Syddall H.E., Cooper R., Benzeval M., Deary I.J., Dennison E.M., Der G., Gale C.R., Inskip H.M., Jagger C. (2014). Grip Strength across the Life Course: Normative Data from Twelve British Studies. PLoS ONE.

[18] Buchard B., Boirie Y., Cassagnes L., Lamblin G., Coilly A., Abergel A. (2020). Assessment of Malnutrition, Sarcopenia and Frailty in Patients with Cirrhosis: Which Tools Should We Use in Clinical Practice?. Nutrients.

[19] García S., Monserrat-Mesquida M., Ugarriza L., Casares M., Gómez C., Mateos D., Angullo-Martínez E., Tur J.A., Bouzas C. (2025). Ultra-Processed Food Consumption and Metabolic-Dysfunction-Associated Steatotic Liver Disease (MASLD): A Longitudinal and Sustainable Analysis. Nutrients.

[20] Gerontiti E., Shalit A., Stefanaki K., Kazakou P., Karagiannakis D.S., Peppa M., Psaltopoulou T., Paschou S.A. (2024). The Role of Low Glycemic Index and Load Diets in Medical Nutrition Therapy for Type 2 Diabetes: An Update. Hormones.

[21] Kawaguchi T., Taniguchi E., Itou M., Sakata M., Sumie S., Sata M. (2011). Insulin Resistance and Chronic Liver Disease. World J. Hepatol..

[22] Marchesini G., Moscatiello S., Domizio S.D., Forlani G. (2008). Obesity-Associated Liver Disease. J. Clin. Endocrinol. Metab..

[23] Temmerman F., Missiaen L., Bammens B., Laleman W., Cassiman D., Verslype C., Pelt J.V., Nevens F. (2011). Systematic Review: The Pathophysiology and Management of Polycystic Liver Disease. Aliment. Pharmacol. Ther..

[24] Aussilhou B., Dokmak S., Dondero F., Joly D., Durand F., Soubrane O., Belghiti J. (2018). Treatment of Polycystic Liver Disease. Update on the Management. J. Visc. Surg..

[25] Kothadia J.P., Kreitman K., Shah J.M. (2023). Polycystic Liver Disease.

[26] Starzl T.E., Reyes J., Tzakis A., Mieles L., Todo S., Gordon R. (1990). Liver Transplantation for Polycystic Liver Disease. Arch. Surg..

[27] Dan A.A., Younossi Z.M. (2006). Quality of Life and Liver Transplantation in Patients with Polycystic Liver Disease. Liver transplantation: Official publication of the American Association for the Study of Liver Diseases and the International Liver Transplantation Society. Liver Transplant..

[28] Takakusagi S., Masuda Y., Takagi H., Yokoyama Y., Kizawa K., Marubashi K., Kosone T., Soejima Y. (2022). Massive Polycystic Liver with a Poor Performance Status Successfully Treated by ABO-Incompatible Adult Living-Donor Liver Transplantation While Overcoming Complications. Intern. Med..

[29] van Aerts R.M.M., van de Laarschot L.F.M., Banales J.M., Drenth J.P.H. (2018). Clinical Management of Polycystic Liver Disease. J. Hepatol..

[30] Merli M., Berzigotti A., Zelber-Sagi S., Dasarathy S., Montagnese S., Genton L., Plauth M., Parés A. (2019). EASL Clinical Practice Guidelines on Nutrition in Chronic Liver Disease. J. Hepatol..

[31] Anand A.C. (2017). Nutrition and Muscle in Cirrhosis. J. Clin. Exp. Hepatol..

[32] Vell M.S., Creasy K.T., Scorletti E., Seeling K.S., Hehl L., Rendel M.D., Schneider K.M., Schneider C.V. (2023). Omega-3 Intake Is Associated with Liver Disease Protection. Front. Public Health.

[33] Zelber-Sagi S., Bernadette Moore J. (2024). Practical Lifestyle Management of Nonalcoholic Fatty Liver Disease for Busy Clinicians. Diabetes Spectrum..

[34] Osna N.A., Donohue T.M., Kharbanda K.K. (2017). Alcoholic Liver Disease: Pathogenesis and Current Management. Alcohol Res. Curr. Rev..

[35] Matsubara T., Sato Y., Igarashi S., Matsui O., Gabata T., Nakanuma Y. (2014). Alcohol-Related Injury to Peribiliary Glands Is a Cause of Peribiliary Cysts: Based on Analysis of Clinical and Autopsy Cases. J. Clin. Gastroenterol..

[36] Geng Y., Faber K.N., de Meijer V.E., Blokzijl H., Moshage H. (2021). How Does Hepatic Lipid Accumulation Lead to Lipotoxicity in Non-Alcoholic Fatty Liver Disease?. Hepatol. Int..

[37] Bernts L.H.P., Drenth J.P.H., Tjwa E.T.T.L. (2019). Management of Portal Hypertension and Ascites in Polycystic Liver Disease. Liver Int..

[38] Moore K.P., Aithal G.P. (2006). Guidelines on the Management of Ascites in Cirrhosis. Gut.

[39] Plauth M., Bernal W., Dasarathy S., Merli M., Plank L.D., Schütz T., Bischoff S.C. (2019). ESPEN Guideline on Clinical Nutrition in Liver Disease. Clin. Nutr..

[40] Pashayee-Khamene F., Hajimohammadebrahim-Ketabforoush M., Saber-Firoozi M., Hatami B., Naseri K., Karimi S., Ahmadzadeh S., Kord H., Saadati S., Hekmatdoost A. (2022). Salt Consumption and Mortality Risk in Cirrhotic Patients: Results from a Cohort Study. J. Nutr. Sci..

[41] Kumar R., Marrapu S. (2023). Dietary Salt in Liver Cirrhosis: With a Pinch of Salt!. World J. Hepatol..

[42] Haberl J., Zollner G., Fickert P., Stadlbauer V. (2018). To Salt or Not to Salt?-That Is the Question in Cirrhosis. Liver Int. Off. J. Int. Assoc. Study Liver.

[43] Hayes J., Benson G. (2016). What the Latest Evidence Tells Us about Fat and Cardiovascular Health. Diabetes Spectr..

[44] Coniglio S., Shumskaya M., Vassiliou E. (2023). Unsaturated Fatty Acids and Their Immunomodulatory Properties. Biology.

[45] Jia F., Hu X., Kimura T., Tanaka N. (2021). Impact of Dietary Fat on the Progression of Liver Fibrosis: Lessons from Animal and Cell Studies. Int. J. Mol. Sci..

[46] Martin K., Tan S.J., Toussaint N.D. (2021). Total Body Sodium Balance in Chronic Kidney Disease. Int. J. Nephrol..

[47] Capelli I., Lerario S., Aiello V., Provenzano M., Costanzo R.D., Squadrani A., Vella A., Vicennati V., Poli C., Manna G.L. (2023). Diet and Physical Activity in Adult Dominant Polycystic Kidney Disease: A Review of the Literature. Nutrients.

[48] Pahlavani N., Azizi-Soleiman F. (2023). The Effect of Dietary Protein on the Progression of Polycystic Kidney Disease–A Review on Current Evidences. Clin. Nutr. Open Sci..

[49] Ogborn M.R., Sareen S. (1995). Amelioration of Polycystic Kidney Disease by Modification of Dietary Protein Intake in the Rat. J. Am. Soc. Nephrol..

[50] Torres V.E., Grantham J.J., Chapman A.B., Mrug M., Bae K.T., King B.F., Wetzel L.H., Martin D., Lockhart M.E., Bennett W.M. (2011). Potentially Modifiable Factors Affecting the Progression of Autosomal Dominant Polycystic Kidney Disease. Clin. J. Am. Soc. Nephrol..

[51] Igarashi P., Somlo S. (2002). Genetics and Pathogenesis of Polycystic Kidney Disease. J. Am. Soc. Nephrol..

[52] Ko G.J., Rhee C.M., Kalantar-Zadeh K., Joshi S. (2020). The Effects of High-Protein Diets on Kidney Health and Longevity. J. Am. Soc. Nephrol..

[53] Bellar A., Welch N., Dasarathy S. (2020). Exercise and Physical Activity in Cirrhosis: Opportunities or Perils. J. Appl. Physiol..

[54] Macías-Rodríguez R.U., Ruiz-Margáin A., Román-Calleja B.M., Moreno-Tavarez E., Weber-Sangri L., González-Arellano M.F., Fernández-del-Rivero G., Ramírez-Soto K. (2019). Exercise Prescription in Patients with Cirrhosis: Recommendations for Clinical Practice. Rev. Gastroenterol. Mex..

[55] Yoo J., Kim J.E., Kim J., Jeon S., Song Y.-J., Choi K.-H., Sim G., Cho M., Yoon J.-W., Kim H. (2025). Effectiveness of Abdominal Bracing Core Exercises as Rehabilitation Therapy for Reducing Abdominal Symptoms in Patients with Autosomal Dominant Polycystic Kidney Disease and Significant Polycystic Liver Disease. Ren. Fail..

[56] Pirenne J., Aerts R., Yoong K., Gunson B., Koshiba T., Fourneau I., Mayer D., Buckels J., Mirza D., Roskams T. (2001). Liver Transplantation for Polycystic Liver Disease. Liver Transplant..

[57] Taner T., Hilscher M.B., Broda C.R., Drenth J.P.H. (2023). Issues in Multi-Organ Transplantation of the Liver with Kidney or Heart in Polycystic Liver-Kidney Disease or Congenital Heart Disease: Current Practices and Immunological Aspects. J. Hepatol..

[58] Ryu H., Kim H., Park H.C., Kim H., Cho E.J., Lee K.B., Chung W., Oh K.H., Cho J.Y., Hwang Y.H. (2017). Total Kidney and Liver Volume Is a Major Risk Factor for Malnutrition in Ambulatory Patients with Autosomal Dominant Polycystic Kidney Disease. BMC Nephrol..

[59] Trovato F.M., Artru F. (2023). Nutritional Optimization in Liver Transplant Patients: From the Pre-Transplant Setting to Post-Transplant Outcome. Acta Gastro-Enterol. Belg..

